# Modeling nitrogen and phosphorus export with InVEST model in Bosten Lake basin of Northwest China

**DOI:** 10.1371/journal.pone.0220299

**Published:** 2019-07-25

**Authors:** Xu Yang, Guangxing Ji, Chong Wang, Jingping Zuo, Haiqing Yang, Jianhua Xu, Ruishan Chen

**Affiliations:** 1 Key Laboratory of Geographic Information Science (Ministry of Education), East China Normal University, Shanghai, China; 2 Research Center for East-West Cooperation in China, East China Normal University, Shanghai, China; 3 School of Geographic Sciences, East China Normal University, Shanghai, China; 4 School of Social Sciences, Shanghai University of Engineering Science, Shanghai, China; University of the Chinese Academy of Sciences, CHINA

## Abstract

Bosten Lake is an important region of Northwest China that has transformed from a freshwater lake to a saltwater lake since the 1970s. The water quality in the Bosten Lake basin is important for social and economic development, and nitrogen (N) and phosphorus (P) are the key indicators of water quality. The land use data, precipitation data and Digital Elevation Model (DEM) data with the Integrated Valuation of Ecosystem Services and Tradeoffs (InVEST) model were used to simulate the N and P exports of the Bosten Lake basin. The spatial and temporal dynamics of nitrogen and phosphorus exports, and the response of nitrogen and phosphorus exports to land use change and precipitation change were analyzed between 2000 and 2015. The results show that the amount of N and P exports increased during 2000–2015, and the N and P exports are mainly distributed around Bosten Lake. The N and P exports are greatly affected by cultivated land, built-up areas and grassland, while they are less affected by other land use types. The high precipitation areas with small exports of N and P are mainly distributed in mountain areas, while small precipitation areas with large exports of N and P are distributed in plains where the cultivated land and built-up areas are concentrated. The InVEST model can be used in Northwest China, and the statistical downscaling of reanalysis precipitation data can be used in the InVEST model to improve the simulation accuracy in the data scarce regions of Northwest China.

## Introduction

The water shortage in the arid regions of the world is the most severe [1,2]. Water resources are the key factors that constrain socioeconomic development and ecological security in the arid regions of Northwest China and play vital roles in the sustainable development of the region [3,4]. The water resources in the arid regions are intertwined with the local livelihoods and the extremely fragile ecological environments, resulting in a contradiction between ecological protection and economic development [5]. At the same time, the arid mountain regions in Northwest China are also the sources of many international rivers. The instability of water resources will cause new problems among the neighboring countries of Central Asia and become a focus of international attention.

Water quality assessment is a hotspot in the field of hydrology and watershed management [6–7]. The studies on water quality assessment mostly use mathematical methods combined with measured data [8–10], but it is difficult to complete the work in some studies because of lack of measured data [11]. In recent years, with the development of Geographic Information System (GIS) and Remote Sensing (RS), models such as Soil and Water Assessment Tool (SWAT) [12], Hydrological Simulation Program Fortran (HSPF) [13] and Integrated Valuation of Ecosystem Services and Tradeoffs (InVEST) [14] have been developed and used to evaluate water quality. The InVEST model is the most widely used tool because of its simple, rapid and strong spatial expression, especially for quantifying ecosystem services in areas with fewer data. The Nutrient Delivery Ratio (NDR) module in the model is specifically used for the simulation of nitrogen and phosphorus exports and has been applied in many regions of the world [14–18]. Currently, there are many studies on water quality assessment in Northwest China [19–22]. Most studies on the water quality in the Bosten Lake basin have mainly focused on Bosten Lake [23–25]. Yu [24] collected 13 samples of surface sediment in Bosten Lake and analyzed total organic carbon (TOC), total nitrogen (TN), stable carbon isotopic composition in TOC, and grain size. Their study indicated that the spatial distribution of sediment TOC in the Bosten Lake was influenced by multiple and complex processes. However, they ignore the influence of water quality between Bosten Lake and its surrounding areas, and there is limited research on the response of nitrogen and phosphorus exports to land use change and precipitation change in the Bosten Lake basin.

In this paper, the InVEST model was used to simulate the nitrogen and phosphorus exports of the Bosten Lake basin. The spatial and temporal dynamics of nitrogen and phosphorus exports, and the response of nitrogen and phosphorus exports to land use change and precipitation change were analyzed between 2000 and 2015. This study is helpful for understanding the water quality of the Bosten Lake basin from the perspective of nitrogen and phosphorus, and provides a theoretical basis for water resource management.

## Study region

### Overview of the study region

**Fig 1:**
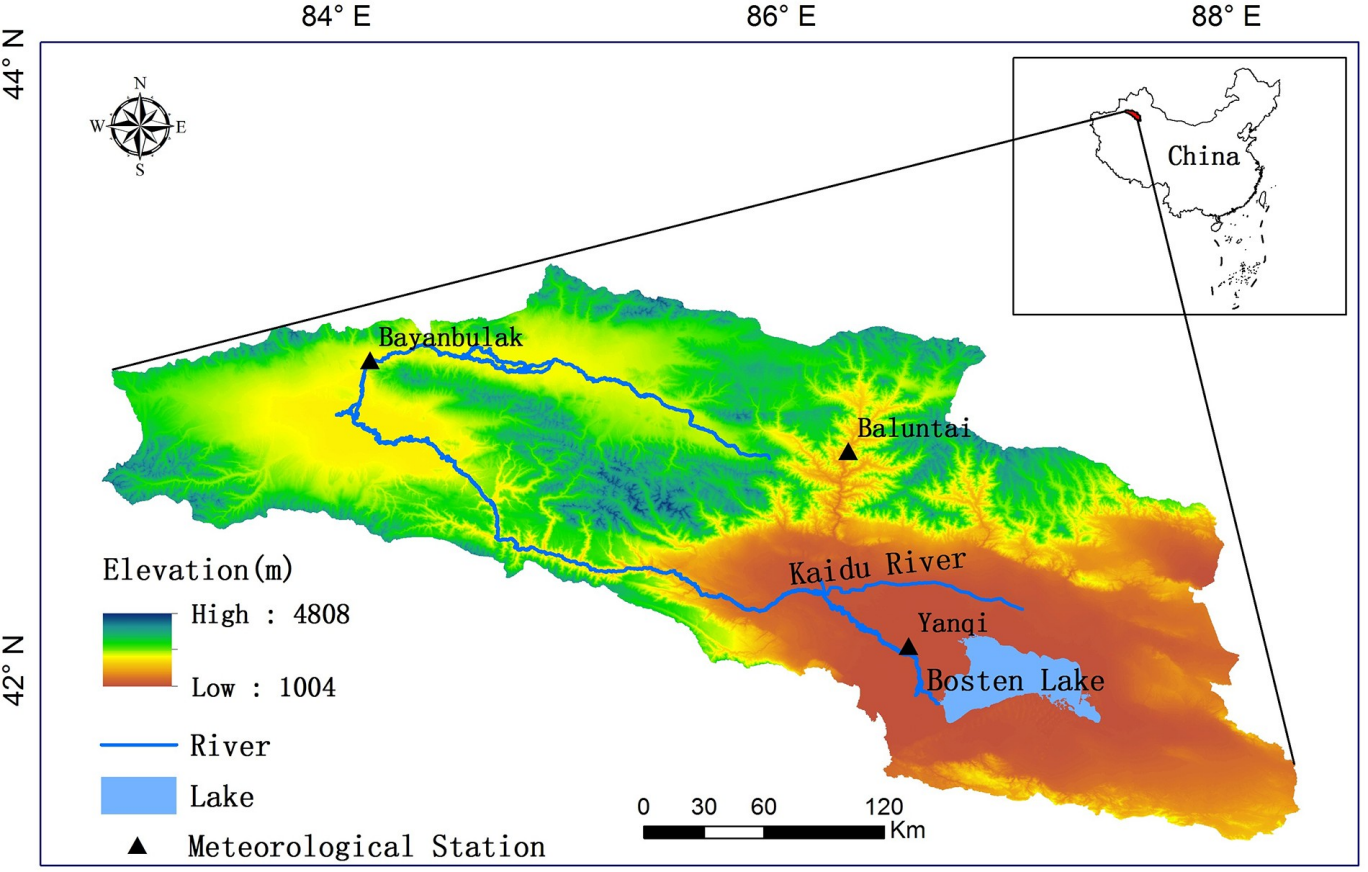
The geographical location of the Bosten Lake basin in Northwest China.

The Bosten Lake basin is an important inland river basin. It is located on the northern edge of the Yanqi Basin and in the southern part of the Tianshan Mountains in the Xinjiang Uyghur Autonomous Region of China, which is between E82°58'~86°05', N42°14'~43°21' (Fig 1), including the Kaidu River, Bosten Lake and other geographic units. The Kaidu River originates in the middle part of the basin. The upstream portion of the Kaidu River originates in the Bayanbulak Grassland and then flows through the Yanqi Basin [26] and finally injects into Bosten Lake, which is the largest inland freshwater lake in China [27]. The Kaidu River has a total length of 560 km, and the total area of the Bosten Lake basin is 44019.07 km^2^. The basin is deeply located in the hinterland of Eurasia, which has a temperate continental arid climate with abundant sunshine and strong solar radiation. The average annual temperature is between 8 °C and 8.6 °C, the annual precipitation is between 70 mm and 600 mm, and the evaporation is between 500 mm and 1500 mm. It has a large elevation difference from north to south, which can be roughly divided into three parts: the semiarid and semihumid areas of the high, cold Tianshan in the northern part of the basin with an altitude of 2500 m to 5000 m; the hilly area and the Gobi desert areas in the central part of basin with an altitude of 1500 m to 2500 m; and the temperate arid zones of the Gobi Plain in the southern part of the basin with an altitude of 1000 m to 1500 m [28].

### Data source

The data used in this analysis mainly include the digital elevation model (DEM), land use data and precipitation data. The ASTER GDEM data were downloaded from the United States Geological Survey (USGS) (https://earthexplorer.usgs.gov). The 30 m resolution data cover land areas between 83°N and 83°S, reaching 99% of the land surface on the earth.

The Landsat TM/ETM data, which were downloaded from National Aeronautics and Space Administration (NASA) (https://www.nasa.gov/), were interpreted according to the classification system of land use [29, 30]. To obtain 30 m resolution land use data in 2000, 2005, 2010, and 2015, we combined the land use data from NASA and the remote sensing information from Google Earth. The average classification accuracy of cultivated land and built-up areas is over 85%, and the average classification accuracy of other land use types is more than 75%.

Due to only three meteorological observation stations in the study area, the spatial resolution of the measured precipitation is obviously very low if used as the input for the precipitation dataset. Therefore, we used the reanalysis precipitation data from the Tropical Rainfall Measuring Mission satellite (TRMM) from NASA (https://www.nasa.gov/). After analyzing and comparing according to previous literature [31–33], the statistical downscaling method was used to deal with reanalysis precipitation data, and then, the statistical downscaling of precipitation data was used as the input data for further use.

The input data of the InVEST model included the biophysical table, threshold flow accumulation, Borselli k parameter, subsurface maximum retention efficiency of nitrogen and phosphorus, and subsurface critical length of nitrogen and phosphorus. The biophysical table includes the output load factor, the maximum retention efficiency, and the critical length of nitrogen and phosphorus for each land use type. Since there were no data on load factor, retention efficiency, and critical length of nitrogen and phosphorus in the Bosten Lake basin, the relevant parameters were selected according to sensitivity analysis based on the similarity of the environment. To run the model, we set the parameters according to the InVEST User’s Guide and the relevant regional literature [34–36]. The subsurface retention efficiency was 0.8, the subsurface critical length was 200 m, the threshold flow accumulation was 1000 for 30 m resolution DEM, and the Borselli k parameter value was 2.

## Methods

**Fig 2:**
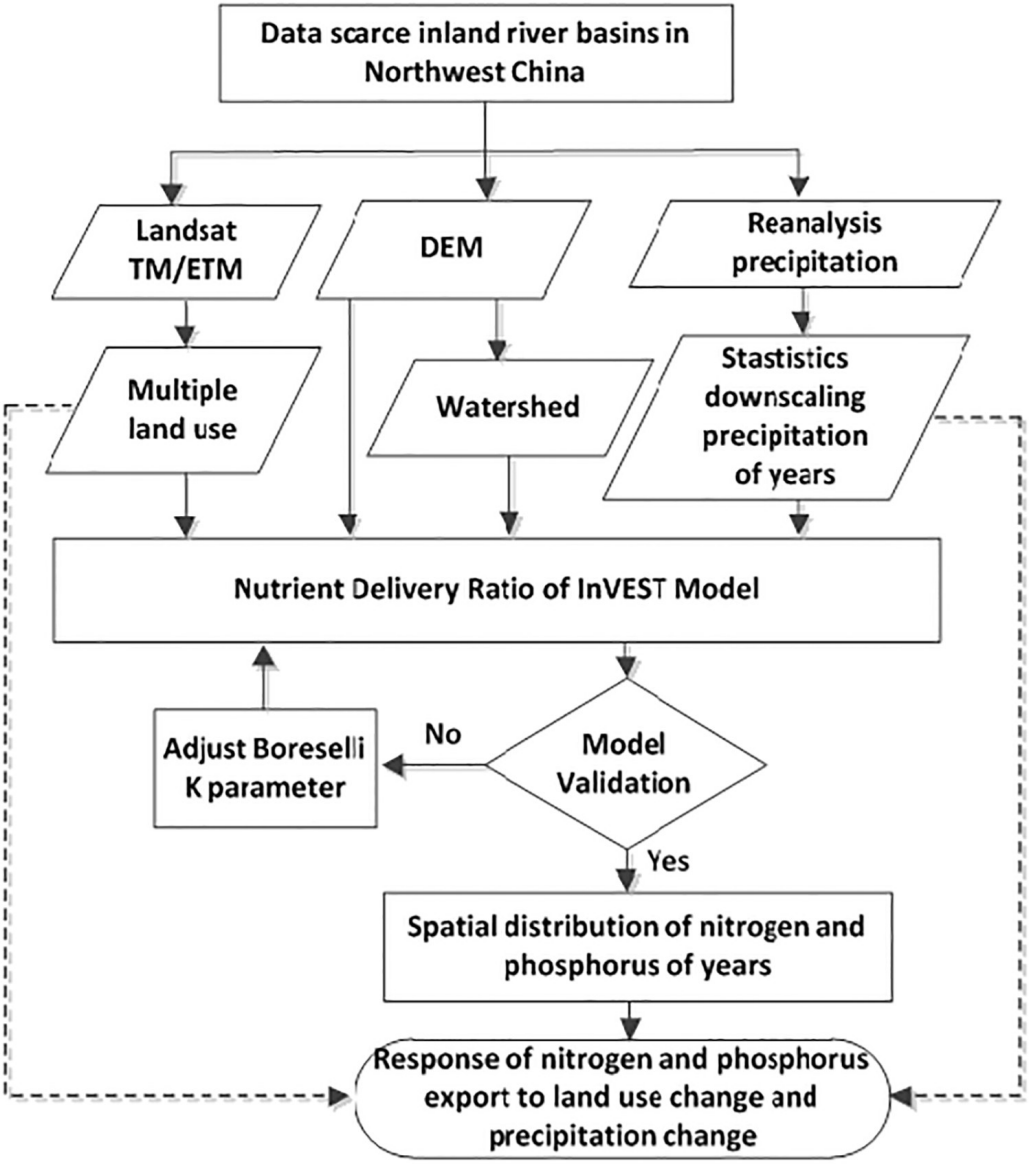
The research framework and method used the NDR module of the InVEST Model.

In this paper, the water quality assessment was based on statistical downscaling of reanalysis precipitation and the InVEST model. First, a statistical downscaling method for reanalysis precipitation data was used to obtain high-resolution precipitation data, which were then inputted into the InVEST model along with DEM, land use and other data to simulate the nitrogen and phosphorus exports in the Bosten Lake basin of Northwest China. The research framework and method are shown in Fig 2.

### Statistical downscaling of reanalysis precipitation data

The spatial precipitation variability is related to altitude. With the terrain data, the regional space-time climate characteristics can be revealed by downscaling of the reanalysis data [37,38]. An improved downscaling method that can dynamically adjust the parameter based on terrain features [39] was applied to downscale the reanalysis climate data. The downscaling method includes three steps: precipitation gradient simulation, reanalysis climate data downscaling and high-resolution precipitation data calculating.

The average monthly precipitation *p*_*i*_ was calculated first to reduce the impact of parameter error on downscaling:

$$p_{i}=\sum_{j=1}^{12}{p_{m}}_{ij}/12$$ (1)

where ${p_{m}}_{ij}$ is the month total precipitation in the ith year and jth month.

Existing studies in Northwest China have shown that the altitude h and precipitation p have a quadratic polynomial relationship [39,40]. Therefore, a quadratic polynomial equation *p* = a*h*^2^ + *bh* + *c* was fitted to downscale the reanalyzed precipitation data. The parameters a, b, and c were estimated with meteorological observations. The fitting effect was tested with R^2^ (coefficient of determination) and F Value.

Then, we calculated the precipitation gradient d*p*/d*h* by calculating the derivative of fitting functions:

$$\frac{dp}{dh}=2ah+b$$ (2)

With Eq (2) and changing values of altitude between reanalysis data and ASTER GDEM data, the monthly reanalysis precipitation data were derived. First, based on the altitude of the ASTER GDEM h_s_ and bilinear interpolation resampling method, the elevation of the reanalysis data h_r_ was calculated. To facilitate the matrix operation among different grid sizes, the reanalysis precipitation data p_r_ and h_r_ were resampled to a grid size as in h_s_ by bilinear interpolation. Then, the downscaled monthly precipitation p_d_ with a spatial resolution of 30 m at the elevation h_s_ was obtained by Eq (3):

$$p_{d}=p_{r}+(h_{s}-h_{r})\cdot (\frac{dp}{dh_{s}}+\frac{dp}{dh_{r}})/2$$ (3)

where (*h*_*s*_ − *h*_*r*_) is the change value of elevation, $(\frac{dp}{dh_{s}}+\frac{dp}{dh_{r}})/2$ is the average precipitation gradients between elevation *h*_*s*_ and *h*_*r*_.

With Eq (4), the downscaling equations are as follows:

$$p_{d}=p_{r}+(h_{s}-h_{r})\cdot [a(h_{s}+h_{r})+b]$$ (4)

### Nutrient delivery ratio of the InVEST model

The Nutrient Delivery Ratio (NDR) of the InVEST model is designed to map the source and transport process of nutrients in a river basin. The spatial distribution of nutrients can be used to assess the retention service of natural vegetation. Retention services play an important role in water quality. The model is mainly used for the simulation of nitrogen and phosphorus. If the recombination rate and permeation rate of the pollutant data are available, the model can also be used for the simulation of other pollutants. The NDR model uses the mass conservation method to simulate the transfer of nutrients in space. Unlike other complex nutrient models, the model does not depict the details of the nutrient cycle and simulates the long-term stable movement of nutrients.

The NDR model estimates the total removal amount of pollutants of nitrogen and phosphorus in the runoff by vegetation and soil and the final export amount of total nitrogen and total phosphorus of the grid to reflect its contribution to water purification [17,41]. The model calculation principle is as follows:

$$X_{expton}=\sum_{i}X_{expi}$$ (5)

$$X_{expi}={load}_{surf,i}\cdot {NDR}_{surf,i}+{load}_{subs,i}\cdot {NDR}_{subs,i}$$ (6)

In the formula, X_expton_ is the total export amount of nutrients in the river basin (kg·yr^-1^), and X_expi_ is the export amount of each grid of nutrients (kg·yr^-1^). The load_surf,i_ is the surface nutrient load (kg·ha^-1^·yr^-1^), NDR_suf,i_ is the surface nutrient transfer rate, load_subs,i_ is the subsurface nutrient load (kg·ha^-1^·yr^-1^), NDR_subs,i_ is the subsurface nutrient transfer rate.

## Results

### Distribution and change of land use from 2000 to 2015

The land use types in the Bosten Lake basin include cultivated land (CL), forest land (FL), grassland (GL), water areas (WA), built-up areas (BA), and unused land (UL). From 2000 to 2015 (Table 1), 50% of the total area of the Bosten Lake basin was grassland, and 30% of it was unused land. The cultivated land accounts for approximately 4–5% of the total area. The forest land is very limited, with approximately 1% of the total area. The built-up areas accounted for less than 0.5% of the total area and were the least common land use type.

**Table 1:** Areas and percentages of land use types in 2000, 2005, 2010 and 2015.

Land use types	2000		2005		2010		2015	
	Area/km^2^	Percent/%	Area/km^2^	Percent/%	Area/km^2^	Percent/%	Area/km^2^	Percent/%
**CL**	1817.73	4.13	1883.94	4.28	2466.77	5.60	2550.54	5.79
**FL**	381.56	0.87	395.50	0.90	363.48	0.83	368.62	0.84
**GL**	21800.62	49.53	20864.94	47.40	24890.91	56.55	24486.94	55.63
**WA**	5270.07	11.97	5241.94	11.91	3468.19	7.88	3456.25	7.85
**BA**	157.40	0.36	196.54	0.45	229.94	0.52	263.43	0.60
**UL**	14591.71	33.15	15436.21	35.07	12599.78	28.62	12893.29	29.29

**Fig 3:**
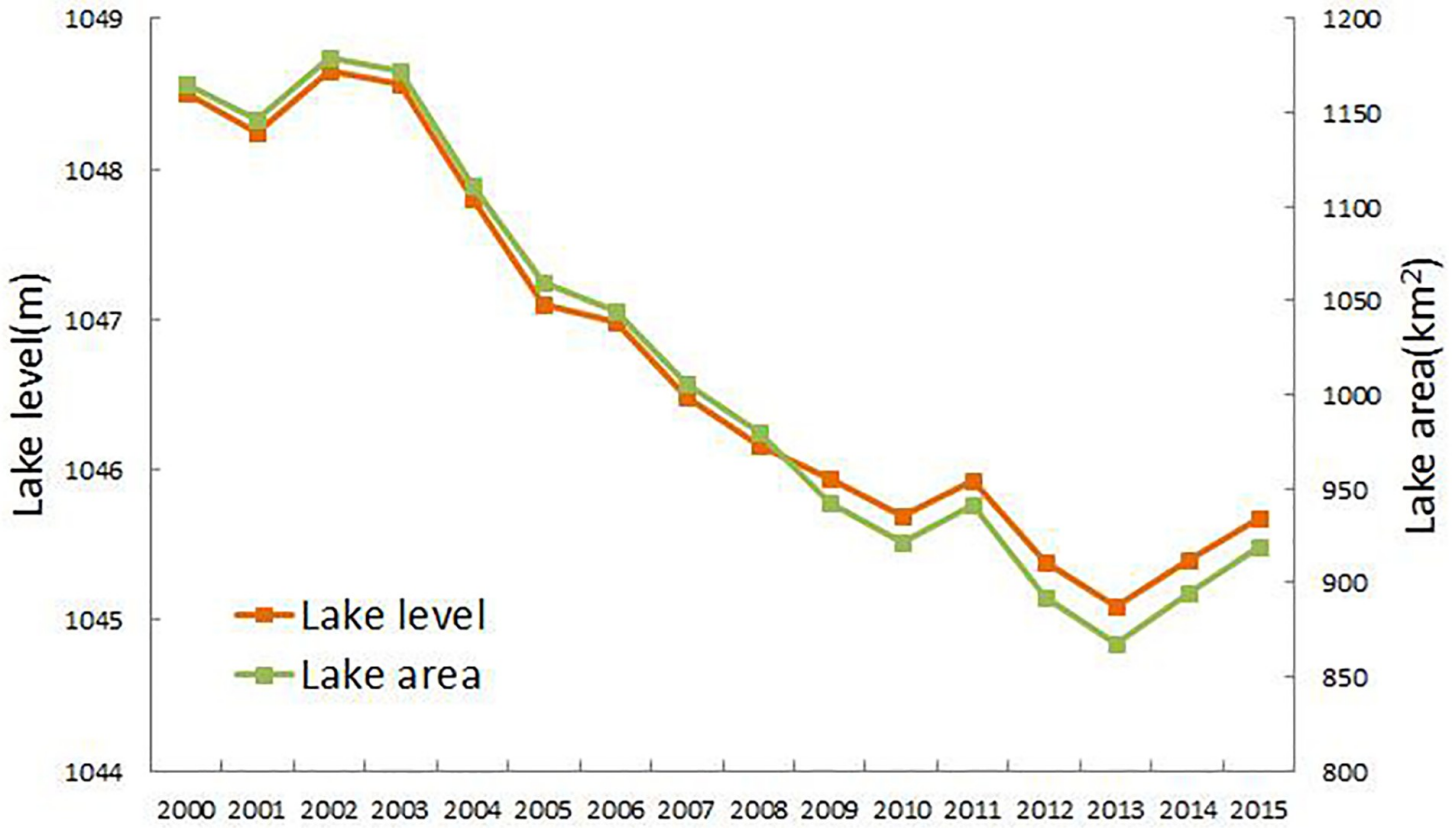
Changes in lake level and lake area of Bosten Lake during 2000–2015.

The distribution of the land use types shows spatial heterogeneity. The cultivated land is mainly distributed around Bosten Lake. In addition, it increases steadily, mainly in the periphery of the oasis, because of irrigation expansion. The water areas decreased rapidly, with a 5% decline in 15 years. It has been reported that Bosten Lake’s area and level had a drastic decrease between 2003 and 2012 and increased in 2013[42,43], as shown in Fig 3. This change is mainly caused by reducing river discharge and increasing water use in agriculture and cities. In addition, the built-up areas increased from less than 0.2% to approximately 0.4% during the study period, nearly doubling because of rapid urbanization.

### Precipitation changes from 2000 to 2015

The meteorological stations including Baluntai, Bayinbulak, Yanqi, Korla, Kumish, and Bugur were selected to check the results of the statistical downscaling of precipitation. The statistical downscaling results are basically consistent with meteorological station data, and the error is small. The quadratic function relationship between precipitation and altitude was strong (R^2^ = 0.648) and significant (F = 94.101, a = 0.005).

**Fig 4:**
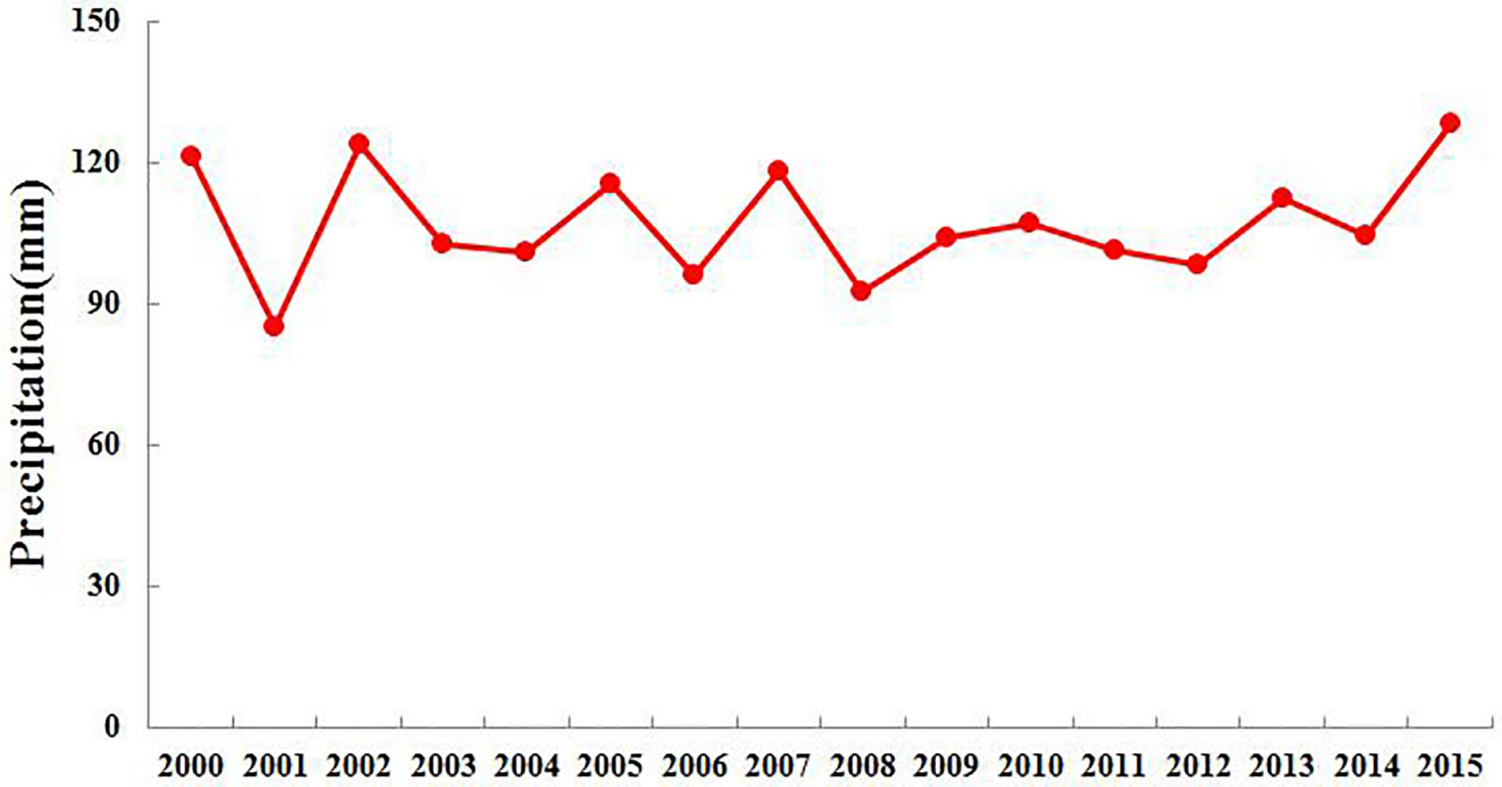
The annual average precipitation of the whole Bosten Lake basin between 2000 and 2015.

**Fig 5:**
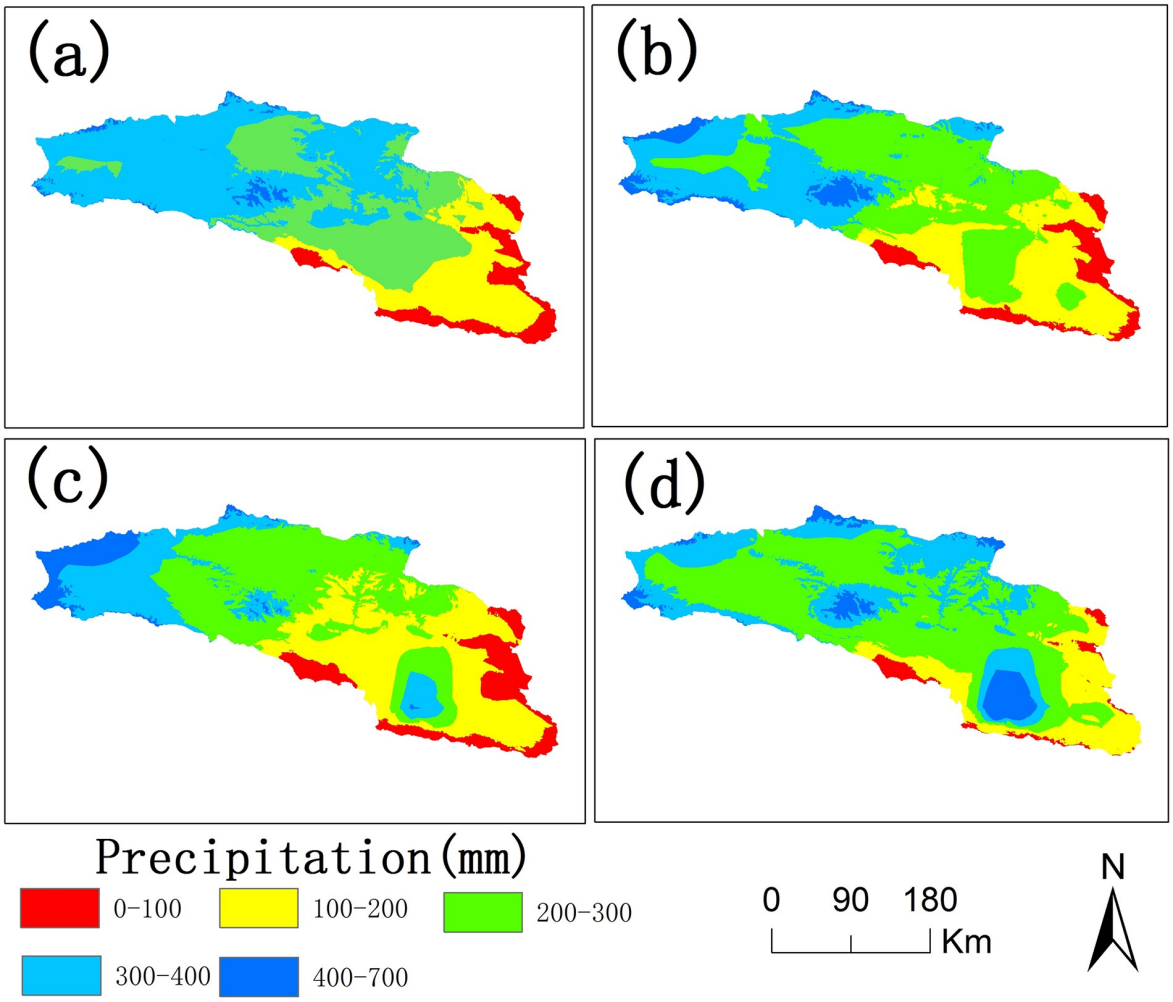
The precipitation of the Bosten Lake basin in 2000(a), 2005(b), 2010(c), 2015(d).

The annual average precipitation of the whole Bosten Lake basin was between 80 mm and 135 mm from 2000 to 2015 in Fig 4. It had a decreasing trend in the decades of 2000 to 2010 and started to increase after 2010[42], as shown in Fig 4. The distribution of precipitation in the Bosten Lake basin is highly uneven. Due to the scarcity of observed meteorological stations, the statistical downscaling method was used to improve the resolution of precipitation data from the reanalysis precipitation data from 2000, 2005, 2010 and 2015 (Fig 5). The precipitation is low in the lower river basin, especially the plains, but it is nearly 10 times higher in the upper river basin, where the elevation is primarily 2500 meters above mean sea level.

### The impacts of land use and precipitation changes on N and P exports

The exports of nitrogen and phosphorus contributed to the increasing total dissolved solids and eutrophication of Bosten Lake. During most of the study period, the water quality of Bosten Lake and its input rivers were Type III to IV water in China’s water quality standards, which means it was not safe to drink directly [44–46]. In China’s water quality standards, the Ministry of Ecology and Environment of the People’s Republic of China promulgated “the standards of surface water environmental quality in China” in 2002 as unpolluted, slightly or moderately polluted (Grades I–III), polluted (Grade IV), and heavily polluted (Grades V and V+) water qualities are shown. In terms of utilities, Grade V+ is not suitable for any use, whereas Grades II-V can be used for restricted purposes (e.g., rare and valuable aquatic species, swimming and aqua farming, industrial and recreational activities that do not include direct contact with human bodies, and agricultural uses and landscape design, respectively).

**Fig 6:**
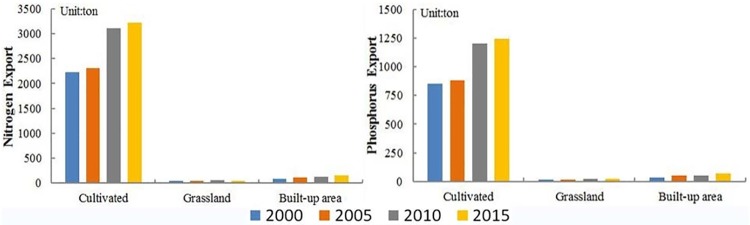
The nitrogen and phosphorus export of cultivated land, grassland and built-up areas in 2000, 2005, 2010 and 2015.

The total exports of nitrogen and phosphorus in 2000 was 2374.92 t and 929.38 t, respectively, which increased to 3439.92 t and 1354.62 t in 2015. Cultivated land is the main source of the exports of nitrogen and phosphorus. The nitrogen and phosphorus exports on cultivated land account for over 90% of the total amount, and the nitrogen and phosphorus exports on grassland and built-up areas are large in the remaining proportion (Fig 6). However, the nitrogen and phosphorus exports on forest land, water areas, and unused land are very few, hardly affecting the export of nitrogen and phosphorus in the Bosten Lake basin. The results of the study region are consistent with existing studies in the Bosten Lake basin. It has been reported that 4.8×10^8^ m^3^ of polluted water is exported from cultivated land to Bosten Lake, accounting for 96% of the total wastewater flows to Bosten Lake and yielding approximately 2700 t N and 260 t P in 2007 [46]. The N and P had a rapid increase from 2000 to 2015, with nearly 0.7 mg/L and 12 ug/L in 2000 to 1.0 mg/L and 32 ug/L in 2010, respectively. As a result, the total dissolved solids increased from 1200 mg/L in 2000 to 1500 mg/L in 2010 [42,46]. The nitrogen and phosphorus exports from different land use types showed different trends over the study period. They increased on the cultivated land and built-up areas from 2000 to 2015, and they decreased on grassland during 2000–2005 and 2010–2015 and increased during 2005–2010 (Fig 6).

The range of annual precipitation is too large to be classified separately in the Bosten Lake basin. We reclassified the precipitation dataset into 5 intervals with 0~100 mm, 100~200 mm, 200~300 mm, 300~400 mm, and 400~700 mm. The annual precipitation in the study region is mainly between 100 mm and 300 mm because the annual precipitation in the arid regions of Northwest China is very low [47].

**Fig 7:**
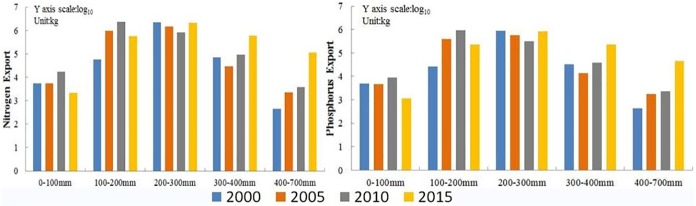
The nitrogen and phosphorus exports for different precipitation intervals in the Bosten Lake basin.

The precipitation had an impact on the nitrogen and phosphorus exports in the basin. From 2000–2015, the amount of nitrogen and phosphorus exports were largely between 100 mm and 300 mm, less than 100 mm or above 400 mm (Fig 7). The high precipitation regions with a small amount of nitrogen and phosphorus exports are mainly in mountain areas northwest of the Bosten Lake basin, where the area of the cultivated land and built-up areas are very small. However, the grassland is mainly distributed in the northwest of the Bosten Lake basin, and nitrogen and phosphorus exports still exist. The low precipitation regions are in the southeast of the Bosten Lake basin, where the cultivated land and built-up areas are distributed, and a large amount of water is used for irrigation, resulting in more nitrogen and phosphorus exports from cultivated land.

### Spatiotemporal distribution and change of N and P

The nitrogen and phosphorus exports continued to grow from 2000 to 2015 in the Bosten Lake basin. They increased by 44.84% and 45.76%, respectively, during 2000–2015 (Table 2).

**Table 2:** The nitrogen and phosphorus exports in 2000, 2005, 2010 and 2015.

Year	Nitrogen export(ton)	Phosphorus export(ton)
**2000**	2374.92	929.38
**2005**	2492.09	971.91
**2010**	3291.47	1291.20
**2015**	3439.92	1354.62

**Fig 8:**
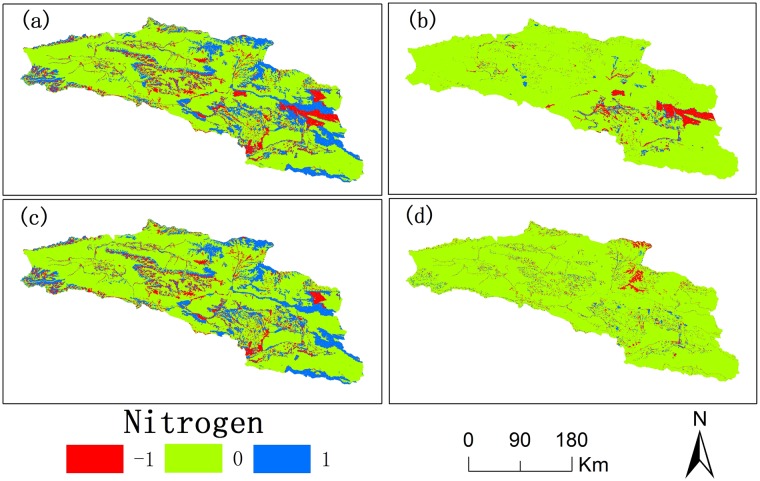
Spatial distribution of the variation in export of nitrogen in the Bosten Lake basin in 2000-2015(a), 2000-2005(b), 2005-2010(c), and 2010-2015(d). (The positive value with 1(blue) indicates the increase in nitrogen export, the negative value with -1(red) indicates the decrease in nitrogen export, and the zero (green) indicates no variation in nitrogen export).

**Fig 9:**
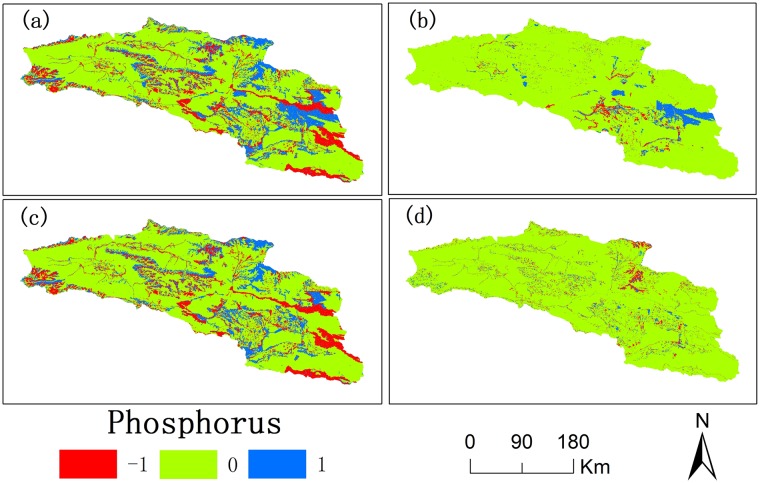
Spatial distribution of the variation in export of phosphorus in the Bosten Lake basin in 2000-2015(a), 2000-2005(b), 2005-2010(c), and 2010-2015(d). (The positive value with 1(blue) indicates the increase in phosphorus export, the negative value with -1(red) indicates the decrease in phosphorus export, and the zero (green) indicates no variation in phosphorus export).

From 2000 to 2015, there was a difference in the spatial distribution of nitrogen and phosphorus exports (Figs 8 and 9). The various amounts of nitrogen and phosphorus exports increased gradually during 2000–2015 (Figs 8a and 9a). The various areas of nitrogen and phosphorus exports first increased and then decreased (Figs 8 and 9). The various regions of nitrogen and phosphorus exports are mainly in the north and west of Bosten Lake where the land use types are cultivated land and built-up areas.

## Discussion

### The trend of N and P exports

**Fig 10:**
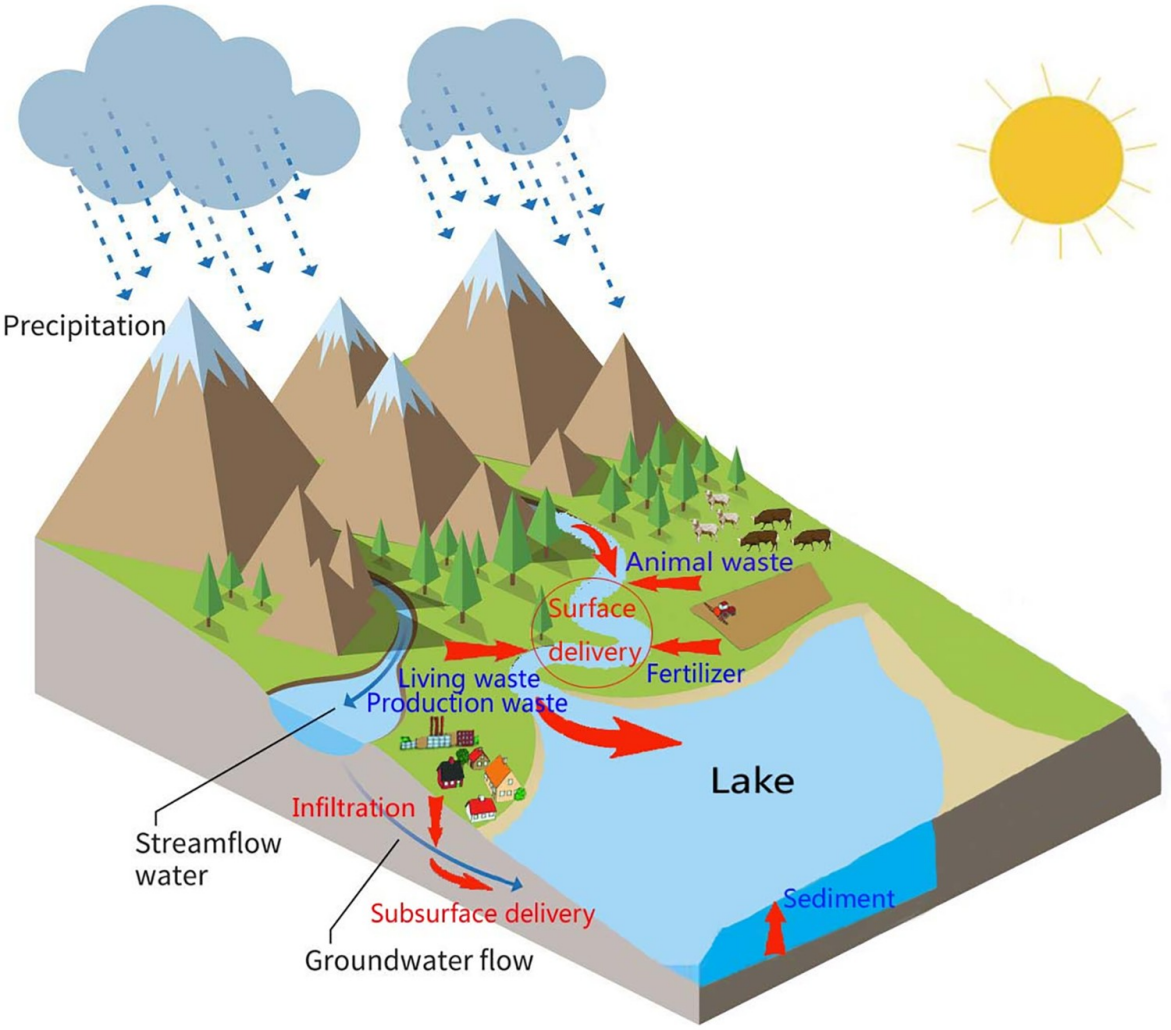
Input source (blue text) and flow direction (red arrow) of nitrogen and phosphorus in the Bosten Lake basin.

Under the influence of high-intensity economic and social activities, the river and lake ecosystems in Northwest China have changed significantly [42]. Pollutants such as nutrients, pesticides, solid waste, aquaculture bait drugs, rural domestic sewage, garbage and settled atmospheric particulate matter enter the water environment through surface runoff and farmland drainage, and they cause nonpoint source pollution and eutrophication [48,49]. Nitrogen and phosphorus are mainly derived from fertilizer, animal waste, production waste, living waste and sediment. Most of the nitrogen and phosphorus enter the surface water and groundwater through surface runoff, erosion, leaching (infiltration or subsurface runoff) and farmland drainage (Fig 10), and they have become important drivers of water quality deterioration in groundwater, rivers and lakes. However, the nitrogen and phosphorus in the Bosten Lake basin are mainly derived from cultivated land and built-up areas, which cause a large amount of pollution, and grassland causes most of the remaining pollution; little of the remaining pollution is caused by the water areas, forest land, and unused land.

The exports of nitrogen and phosphorus in the Bosten Lake basin increased from 2000 to 2015. The environment was very unstable and still faced many challenges in 15 years. The amount of nitrogen and phosphorus exports in the western and northern parts of Bosten Lake greatly changed where the cultivated land and built-up areas were concentrated (Fig 5). The rapid economic development and population growth of the Bosten Lake basin, such as the agricultural development of the Yanqi Plains [50], has promoted the expansion of cultivated land and built-up areas [51], leading to increasing use of fertilizers and irrigation water and producing more domestic waste.

There was a eutrophication trend in Bosten Lake in recent years [52]; phosphorus is the most important limiting factor [53], and the dissolved oxygen content of the water in Bosten Lake also showed a downward trend [52], making it easier to release phosphorus from sediments into the water [54]. The redox conditions in different dissolved oxygen environments have changed significantly, which in turn affect environmental variables that regulate the release of phosphate, such as active iron [55]. In the release process of phosphorus in the lake, iron-bound phosphorus has potential mobility and is easily dissolved under hypoxic conditions then released by diffusion to the water [56].

### Attribution of N and P pollution and policy implication

The nitrogen and phosphorus mainly come from farmland fertilization, weathering of agricultural waste, domestic sewage in urban or rural areas, industrial wastewater and waste gas. The nitrogen and phosphorus exports were significantly affected by land use and precipitation during 2000–2015 in the Bosten Lake basin, but the response to changes in land use and precipitation was different (Figs 6 and 7). Large amounts of nitrogen and phosphorus exports were mainly produced by cultivated land and built-up areas where the precipitation is low. However, the low amounts of nitrogen and phosphorus exports were in the mountainous areas where the land use types are mainly grassland and unused land where the precipitation is high. It shows that the response of nitrogen and phosphorus exports to land use types is greater than that of precipitation, and it is consistent with the NDR module driven by land use types [14]. To improve the water quality, returning farmland to forests and grasslands and controlling fertilization and grazing are required. The efficiency of fertilizer on cultivated land should be improved to reduce the exports of nitrogen and phosphorus into rivers and Bosten Lake.

To control water pollution and improve water quality in the Bosten Lake basin, the Bayingol Mongolian Autonomous Government, which is affiliated with the Bosten Lake basin, worked with the provincial government and formulated the “Regulations on Water Environment Protection and Pollution Prevention in the Kaidu River and Kongque River Basin of Bayingol Mongolian Autonomous Prefecture" in 2017 and the “Implementation Plan for Protection and Governance of the Water Environment with the Joint Action of Government and Force in the Bosten Lake Basin” in 2018. The targets of these regulations are improving the water quality in Bosten Lake to Type III water in 2020.

### Scenarios of water quality

Currently, the population expansion in the Bosten Lake basin has led to the development of agriculture and grazing, which has led to an increase in the area of cultivated land, grassland and built-up areas [51], resulting in excessive fertilizer, livestock manure pollution, production and living wastes that will increase the amount of nitrogen and phosphorus. Social and economic development of the basin will destroy forest land and grassland and exploit unused land to meet the expansion of cultivated land and built-up areas. The reduction in natural vegetation and the destruction of soil will eventually lead to a decline in the ability to retain nitrogen and phosphorus. Increased water use for irrigation and livestock, overexploitation of groundwater, and increased sewage discharge have led to water pollution and water area reduction.

From 2000 to 2015, the change of land use showed an increase in cultivated land, grassland, and built-up areas and decreases in forest land, water areas and unused land. The trend is consistent with the state of the Bosten Lake basin, and nitrogen and phosphorus exports will increase if we take no action to protect the water environmental quality in the future, which will cause less available water and less clean water for people in the Bosten Lake basin.

### Limitations and prospects

Although the study provided insights into the assessment of water quality in Northwest China, there is still a large space to improve. The InVEST model has been in use for nearly 10 years at many places around the world, and it is still improving. Some ecological and water-related physicochemical processes are relatively simple and should be considered when interpreting model output [57]. Due to the lack of data, some input parameters are derived from the official user’s guide of the InVEST and literature, and it would be beneficial to improve the simulation results by using the information from the study area to calibrate the input parameters in future research. For example, the nitrogen and phosphorus export rates in this paper were the same during the different research periods; however, these are different because of the changing agricultural structure, soil quality, and technology, and these factors should be examined in detail in the future. The study region does not cover the entire administrative unit. The data for human factors are difficult to match to the biophysical boundaries. In the future, the influence of human factors on water quality should also be considered, such as planting, animal husbandry, aquaculture, industry, construction, tourism, accommodation, and catering industry.

## Conclusions

In this study, the statistical downscaling method was used to obtain high-resolution precipitation data as the input data of the InVEST model to simulate the nitrogen and phosphorus exports in the Bosten Lake basin. The results show that the InVEST model can be applied to the data scarce regions of Northwest China, and the exports of nitrogen and phosphorus generally increased from 2000 to 2015 in the whole basin. The distribution of nitrogen and phosphorus exports are largely in the southeast and are lower in the northwest of the Bosten Lake basin. From the response of nitrogen and phosphorus exports to land use types, there was a great impact on cultivated land, built-up areas and grassland and less impact on forest land, water areas and unused land. From the response of nitrogen and phosphorus exports to precipitation, the low amount of nitrogen and phosphorus exports are in mountainous areas with high precipitation, and the large amount of nitrogen and phosphorus exports are in plain areas with less precipitation where the cultivated land and built-up areas are distributed. In general, nitrogen and phosphorus exports are more responsive to land use types than precipitation.

Rapid urbanization and social and economic development have led to changes in land use and natural environments, which had significant impacts on water resources in Northwest China. Although some measures have been taken, there was still a potential ecological risk. Protection of water resources and natural vegetation, and the rational use of land for sustainable development in Northwest China should be considered.

## Supporting information

S1 Data Dataset. (ZIP)
